# Insect and Mice Infestations in Gaza Displacement Camps: A Field‐Based Study on Vector‐Borne Diseases Amid the 2023–2025 Gaza War

**DOI:** 10.1002/puh2.70127

**Published:** 2025-09-23

**Authors:** Zuhair Dardona, Samia Boussaa

**Affiliations:** ^1^ Governmental Medical Service Gaza Palestine; ^2^ ISPITS‐Higher Institute of Nursing and Technical Health Occupations Ministry of Health and Social Protection Rabat Morocco

**Keywords:** disease vectors, displacement camps, Gaza, infestation, insects, mice, sanitation crisis, war, waste accumulation

## Abstract

The current study investigates the severe environmental and public health impacts resulting from the ongoing conflict in the Gaza Strip, with a particular focus on the rapid proliferation of rodents and insects. Utilizing comprehensive field visits, photographic documentation, and an extensive review of authoritative sources, the research identifies the widespread destruction of critical infrastructure—including sewage systems and potable water networks—as a key driver of this crisis. The resulting accumulation of uncollected waste, rubble from demolished buildings, and stagnant water has created optimal conditions for infestations of flies, mosquitoes, fleas, and notably mice. These infestations pose significant health hazards due to the lack of effective pest control measures, inadequate sanitation, and limited healthcare resources. The study highlights the urgent need for a cessation of hostilities, alongside immediate interventions, such as the restoration of essential water and sanitation services, the introduction of pest control programs, and the provision of medical, hygiene, and nutritional supplies to mitigate the escalating health risks faced by displaced populations.

## Graphical abstract

GRAPHICAL ABSTRACT

Conflict‐driven destruction in Gaza has caused water and sanitation collapse, leading to infestations of rodents and insects, escalating public health risks among displaced populations.

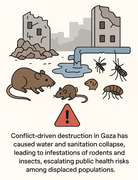

## 1 Introduction

No modern chapter in the region's history has borne witness to devastation as far‐reaching as that unleashed by the protracted war in Gaza, now extending beyond 17 months. The humanitarian consequences have been nothing short of catastrophic. According to data from the Office for the Coordination of Humanitarian Affairs and Gaza's Ministry of Health, by April 2025, the conflict had claimed the lives of over 51,000 Palestinians and left at least 116,343 others wounded [1, 2]. Moreover, over 90% of Gaza's 2.1 million inhabitants have been forcibly displaced, now enduring life in overcrowded shelters plagued by collapsed sanitation infrastructure, the rapid spread of vector‐borne diseases, and acute shortages of food, clean water, and essential medical services [3, 4].

Satellite imagery strikingly illustrates the scale of devastation, indicating that approximately 60% of structures have been either damaged or obliterated—among them, a staggering 92% of residential buildings, 88% of educational facilities, and half of the region's hospitals. This extensive destruction has prompted some estimates to propose that over 10,000 bodies may still lie buried beneath the debris [5]. The widespread destruction of essential infrastructure has precipitated outbreaks of skin infections, hepatitis, and acute malnutrition. Simultaneously, approximately 1.9 million displaced Palestinians endure extreme food insecurity and harsh environmental exposure, residing in makeshift tents that offer little to no protection against the rigors of winter cold or the oppressive summer heat [4, 6, 7]. The prolonged conflict in Gaza (2023–2025) has triggered a devastating humanitarian crisis, with displacement camps becoming focal points for the spread of vector‐borne diseases [8, 9]. The convergence of extreme overcrowding, the collapse of sanitation and sewage systems, and the unchecked accumulation of solid waste has created highly favorable conditions for the rampant proliferation of disease vectors, such as fleas, mosquitoes, as well as important rodent hosts. These factors have significantly intensified the public health burden, deepening the vulnerability and suffering of already displaced and underserved populations [3, 7, 10]. These vectors proliferate in the context of war‐induced infrastructural breakdown, characterized by inoperative sewage systems, contaminated water supplies, and severely disrupted waste disposal mechanisms. Such circumstances have precipitated an alarming surge in skin infections, diarrheal diseases, and zoonotic outbreaks, marking an unprecedented public health emergency in the affected regions [3, 7, 10].

Armed conflicts are widely recognized for fostering conditions that promote the spread of rodents and disease vectors, driven by collapsed sanitation, overcrowding, and waste accumulation. Historical cases—such as the 1943–1945 typhus outbreaks in war‐torn Naples and San Antonio—highlight how displacement and infrastructure breakdown accelerate infestations of *Rattus* spp. and *Xenopsylla cheopis*, the primary vector of flea‐borne typhus, reinforcing the strong association between warfare and the resurgence of vector‐borne diseases [11].

Gaza has been under a crippling blockade since 2007, well before the current conflict. This prolonged siege has severely limited access to the equipment and materials necessary for effective waste management, resulting in recurrent and excessive accumulations of garbage—conditions that are well known to attract rodents and insects. However, the extent of waste‐related hazards reported in the current crisis—such as putrefying piles of refuse and overflowing sewage—appears to be directly associated with the widespread destruction of infrastructure caused by the ongoing war [3, 12].

Despite extensive searches, there is a lack of specific pre‐2023 data on pest populations in Gaza. Available reports tend to focus instead on the war‐induced crisis, emphasizing the devastation caused by bombardments, the deliberate targeting of sanitation infrastructure, and the mass displacement of civilians into overcrowded shelters—all of which have created ideal conditions for pest proliferation. Although structural vulnerabilities existed prior to the conflict, the current surges in rodent and insect infestations are overwhelmingly attributed to the direct consequences of military operations and the prolonged blockade [13, 14, 15]. Given the severity of current conditions, this study critically explores the public health impact of the ongoing conflict in Gaza by investigating insect and rodent infestations in displacement camps, utilizing field observations, humanitarian reports, and relevant academic sources.

## 2 Materials and Methods

### 2.1 The Study Area

**FIGURE 1:**
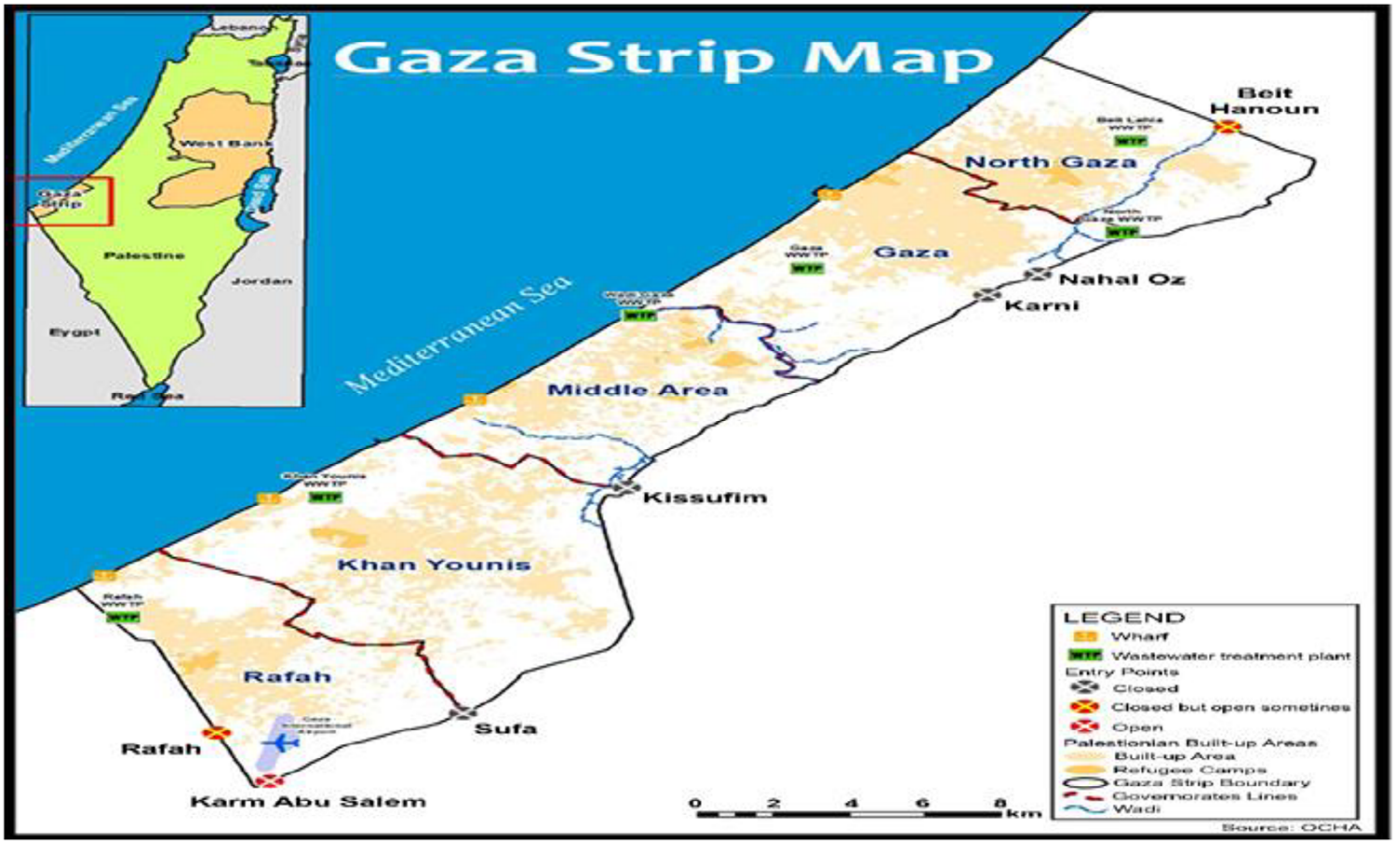
The map of Gaza Strip [20].

The study area includes Gaza's displacement camps, which currently serve as the primary refuge for 1.9 million displaced individuals, constituting 90% of Gaza's population, in the context of Israel's military operations from 2023 to 2025 [16]. Geographically, Gaza's Mediterranean coastal strip spans 365 km^2^, with a pre‐war population density of 6500 people/km^2^—one of the highest globally—now intensified by displacement camps housing up to 88,000 people per square mile in designated “safe zones” like Al‐Mawasi [17, 18]. Demographically, the displaced population comprises approximately 35,055 children who have lost one or both parents and an estimated 48,000 pregnant women experiencing severe food insecurity. These groups are particularly vulnerable to vector‐borne diseases due to the compounding effects of malnutrition, inadequate shelter, and limited access to healthcare [16, 19]. However, Figure 1 illustrates the map of Gaza Strip.

### 2.2 Review Design

The present study adopts a dual‐phase methodology, integrating post‐October 2023 conflict‐related data with pre‐2023 historical baselines to assess vector‐borne risks in Gaza's displacement camps. Priority is given to recent sources—including UN OCHA reports, World Bank damage assessments, and peer‐reviewed analyses—highlighting the impacts of infrastructure collapse, overcrowding, and environmental degradation on vector and rodent proliferation. Pre‐conflict literature on Gaza's environmental health and vector ecology provides essential baseline comparisons. The review includes peer‐reviewed studies and verified humanitarian reports (e.g., UNRWA, ANERA), while excluding non‐empirical and non‐georeferenced sources. This approach allows for the identification of both acute and chronic vulnerabilities contributing to heightened vector‐borne disease risks.

### 2.3 Search Strategy

This study employs a multisource approach to gather data on vector‐borne disease risks in Gaza's displacement camps. Peer‐reviewed literature was retrieved from databases, including PubMed, Scopus, and Google Scholar, supplemented by gray literature from international organizations, such as the World Health Organization (WHO), UNRWA, and local NGOs (e.g., ANERA, Palestinian Medical Relief Society). Search terms included combinations such as “insect fleas,” “mosquitoes,” “rodents,” “Gaza,” “vector‐borne diseases,” and “health risks in displacement camps.” The inclusion criteria focused on studies addressing environmental conditions, disease transmission, and public health impacts—particularly reports published after October 2023, when the escalation of conflict intensified the collapse of sanitation and shelter infrastructure. Special attention was given to verified case updates and situation reports issued by UNRWA, WHO, and other internationally recognized health authorities. It is worth noting that this study was conducted during the period from March to May 2025.

### 2.4 Inclusion/Exclusion Criteria

**Inclusion Criteria**: This review includes studies that address the presence and types of vectors—specifically fleas, mosquitoes, and rodents—as well as the diseases transmitted by these vectors within Gaza's displacement camps following the escalation of conflict in 2023. Eligible studies also examine environmental and infrastructural factors (e.g., overcrowding, poor sanitation, and waste accumulation) that have contributed to the increased proliferation of these vectors during and after the onset of mass displacement.

**Exclusion**: Studies not directly related to the topic or outside the conflict zone context.

### 2.5 Data Extraction

- Extract key information on:
- Vector species and diseases
- Environmental factors (waste, water stagnation)
- Interventions (vector control measures, public health responses)

### 2.6 Data Synthesis

Qualitative synthesis of trends, gaps, and risk assessments related to vector‐borne diseases in Gaza.

### 2.7 Documentations

The present study provides unequivocal evidence of a widespread and escalating infestation of insects, such as flies, mosquitoes, fleas, and lice. This conclusion is based on researchers’ field visits, photographic documentation, and the review of multiple peer‐reviewed studies, statistical reports, and verified websites of international and local organizations, including health and humanitarian agencies.

## 3 Results and Discussion

### 3.1 Insect Infestation

**FIGURE 2:**
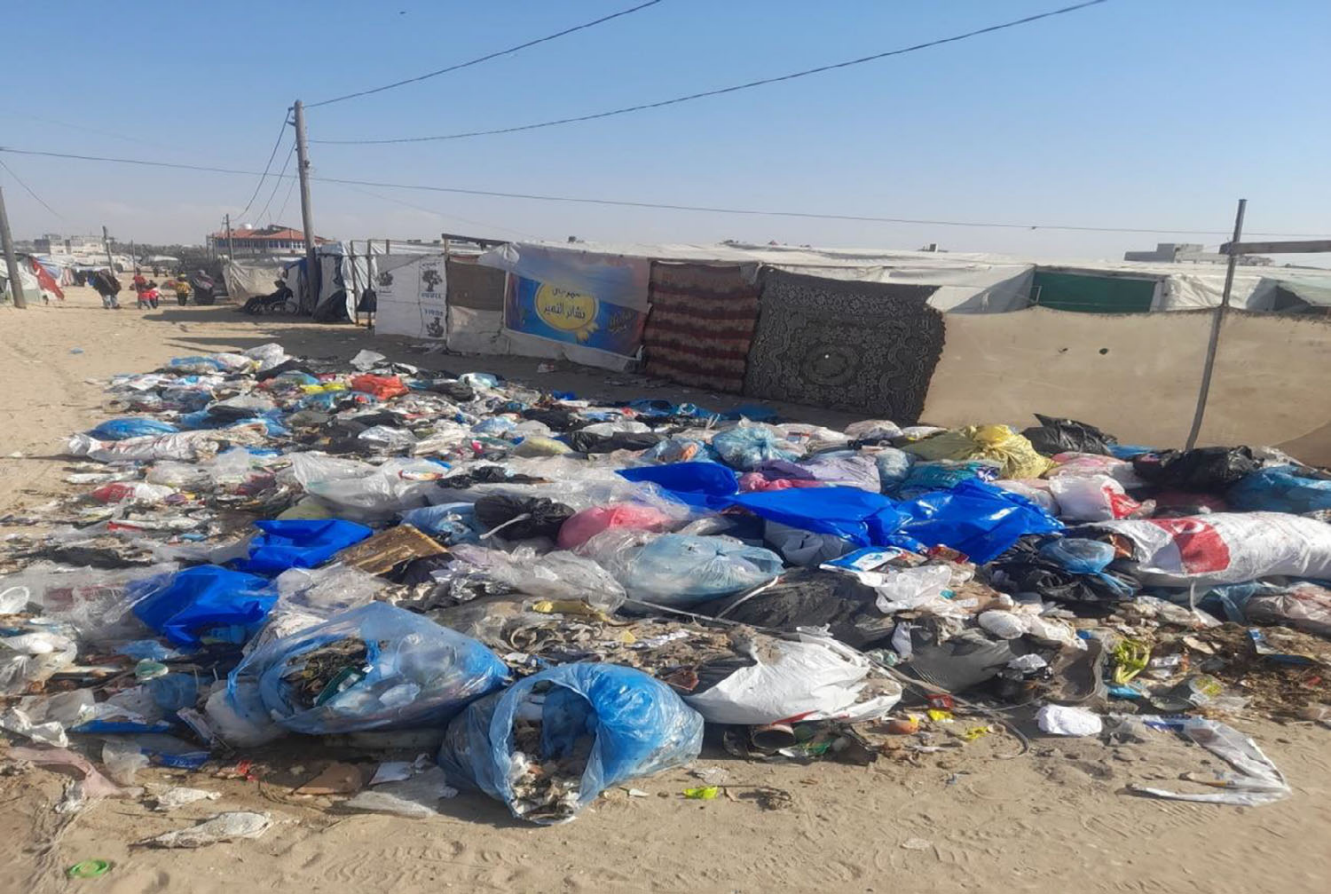
Accumulation of waste adjacent to the tents of displaced persons. *Source:* Photo taken by Dr. Zuhair Dardona.

The present study provides unequivocal evidence—based on researchers’ field visits, photographic documentation, and the review of multiple peer‐reviewed studies, statistical reports, and verified websites of international and local organizations, including health and humanitarian agencies—of a widespread and escalating infestation of insects, such as flies, mosquitoes, fleas, and lice. These vectors have been consistently documented in high and increasing densities among displaced populations in Gaza's displacement camps, particularly in recent months. The destruction of critical infrastructure and public health services as a result of the ongoing conflict has created an environment highly conducive to the proliferation of disease‐carrying insects, particularly flies, mosquitoes, and fleas, in Gaza's displacement camps. Figure 2 illustrates the accumulation of tons of garbage adjacent to tents in displacement camps, forming a fertile breeding ground for insect infestations and posing a serious public health hazard. The collapse of sewage treatment facilities, waste management systems, and access to clean water has led to the uncontrolled accumulation of solid waste and the overflow of untreated sewage in and around displacement areas. These unsanitary conditions have intensified the risk of vector‐borne disease outbreaks among the displaced population.

**FIGURE 3:**
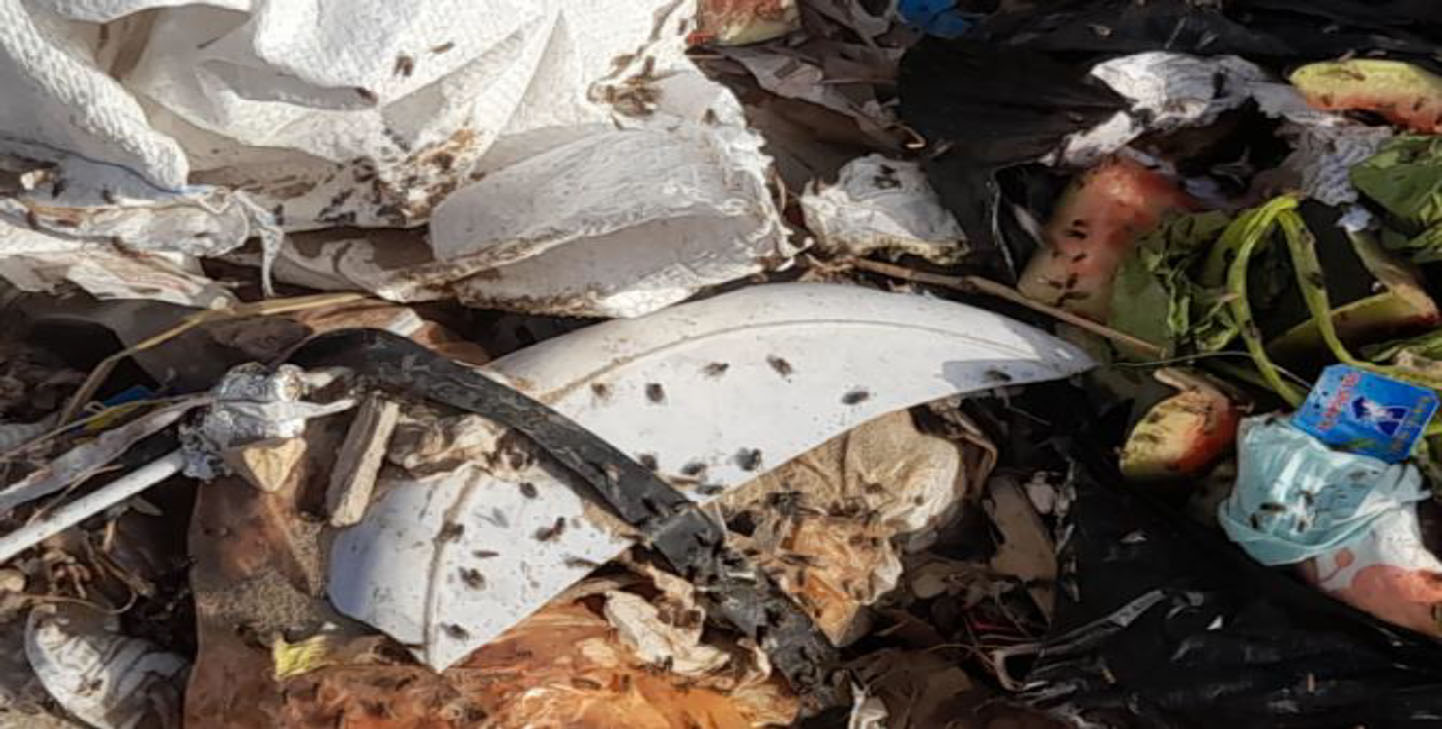
Flies over piles of garbage near tents housing displaced individuals in the displacement camps. *Source:* Photo taken by Dr. Zuhair Dardona.

A well‐documented association exists between the accumulation of solid waste and the proliferation of fly populations. Organic refuse—particularly when improperly managed in open or overflowing disposal sites—creates optimal conditions for the breeding of various fly species [21]. These insects are drawn to the potent odors emitted by decomposing organic material, such as food waste, where they deposit their eggs. Figure 3 shows a dense clustering of flies over piles of garbage near tents housing displaced individuals in the displacement camps. The resulting larvae (maggots) thrive in these moist, nutrient‐dense environments, rapidly developing by feeding on the decaying matter. Empirical studies have demonstrated that even minimal quantities of household waste can give rise to thousands of adult flies within a brief time frame, whereas larger dumpsites or unmanaged landfills can lead to exponential surges in local fly densities [22].

**FIGURE 4:**
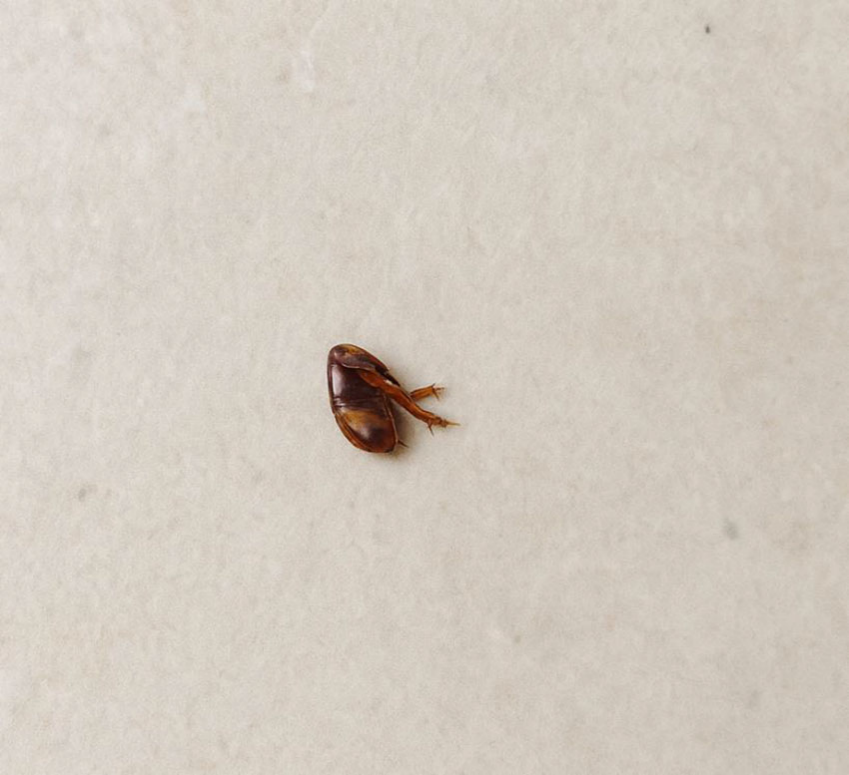
A flea in one of the displacement camps in the Khan Younis Governorate. *Source:* Captured by Dr. Zuhair Dardona.

As is well known, flies are important mechanical vectors for many parasitic, viral, and bacterial pathogens [9]. Among the most significant diseases they can transmit are poliomyelitis and viral hepatitis, not to mention diarrhea and other illnesses. All of these have been documented among displaced individuals in the displacement camps through multiple studies conducted during this war [8, 23]. In addition to flies, the study also documented, through field visits to displacement camps, a severe and widespread infestation of fleas, which intensifies as the camps become more overcrowded. It was observed that the camps with the highest flea infestations also host numerous domestic animals, such as camels, dogs, cats, sheep, and others. Upon detailed investigation, it was found that many displaced persons were compelled to bring their pets with them out of fear for their safety and to protect them from the ravages of war. Figure 4 documents this type of flea infestation prevalent in the displacement camps of the Khan Younis Governorate in the southern Gaza Strip.

Flea infestations in pets result from multiple factors such as contact with infested animals, environmental conditions, and insufficient prevention. Research indicates that households with multiple pets, particularly cats, face a higher risk, with *Ctenocephalides felis* (cat flea) being the most common species affecting both cats and dogs [24]. In addition, warm, humid climates and increased rainfall, especially during late summer, correlate with higher flea infestation rates, as regional studies confirm. Moreover, socioeconomic barriers often impede effective flea control, thereby increasing infestation risks [24, 25]. Fleas may infiltrate households not only through companion animals but also via human vectors transporting eggs or larvae on garments and secondhand goods, in addition to incursions by wildlife or rodent populations. Importantly, the absence or inadequate implementation of prophylactic parasitic control measures constitutes a primary factor in the persistence and recurrence of infestations [26, 27]. The current conflict in Gaza presents a convergence of environmental and socioeconomic stressors, as evidenced by field observations, photographic records, and corroborating reports. Notably, there is an alarming accumulation of solid waste, widespread sewage overflow, and severe overcrowding within displacement camps. These conditions facilitate the proliferation of commensal rodents and domestic animals, including dogs and cats. Furthermore, displaced populations have transported livestock—such as camels and sheep—alongside companion animals, which they regard as critical assets, thereby exacerbating the complexity of managing animal populations and associated health risks amid the ongoing humanitarian crisis. Fleas represent significant public health concerns due to their bites, which induce itching, allergic reactions, and potential secondary infections.

**FIGURE 5:**
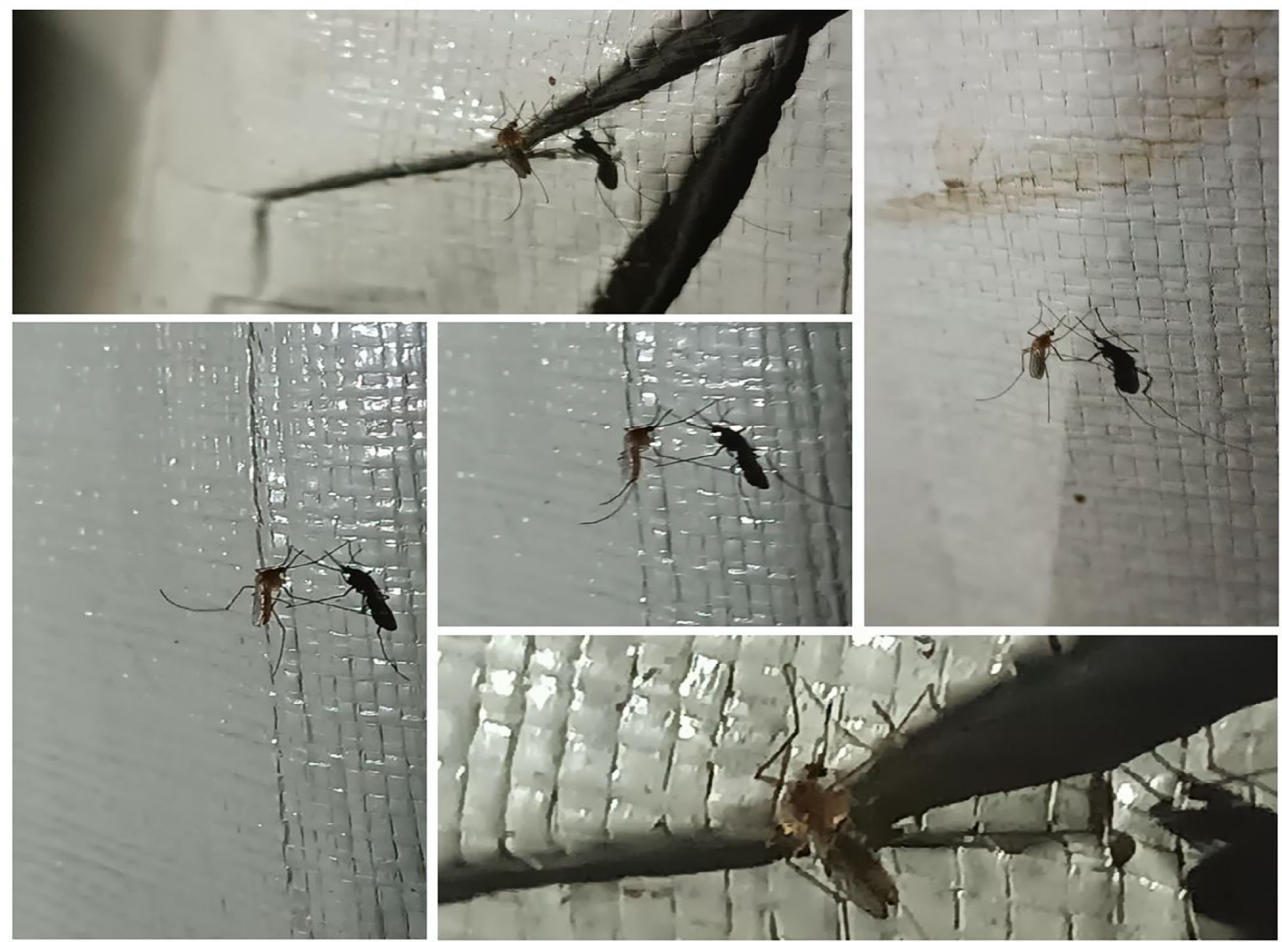
Various mosquito species prevalent in Gaza displacement camps during the current conflict. *Source:* Photo captured by Dr. Zuhair Dardona.

Critically, they serve as vectors for serious diseases such as plague (*Yersinia pestis*), murine typhus (*Rickettsia typhi*), and tapeworm transmission through ingestion of infected fleas. Fleas also facilitate the spread of cat scratch disease (*Bartonella henselae*) from cats to humans. Severe infestations or hypersensitivity can result in complications like anemia and intense allergic dermatitis, particularly in vulnerable individuals [28, 29, 30, 31]. On the other hand, this study documented the alarming and widespread proliferation of mosquitoes in displacement camps and public areas. This infestation was notably concentrated in locations where sewage flooding occurs between tents and near accumulations of waste, creating a significant public health issue that deprives displaced persons of sleep at night [3]. The absence of effective mosquito control measures, due to the ongoing conflict, exacerbates the problem [32]. Figure 5 illustrates some of the mosquito species identified in the displacement camps as documented in this study.

**FIGURE 6:**
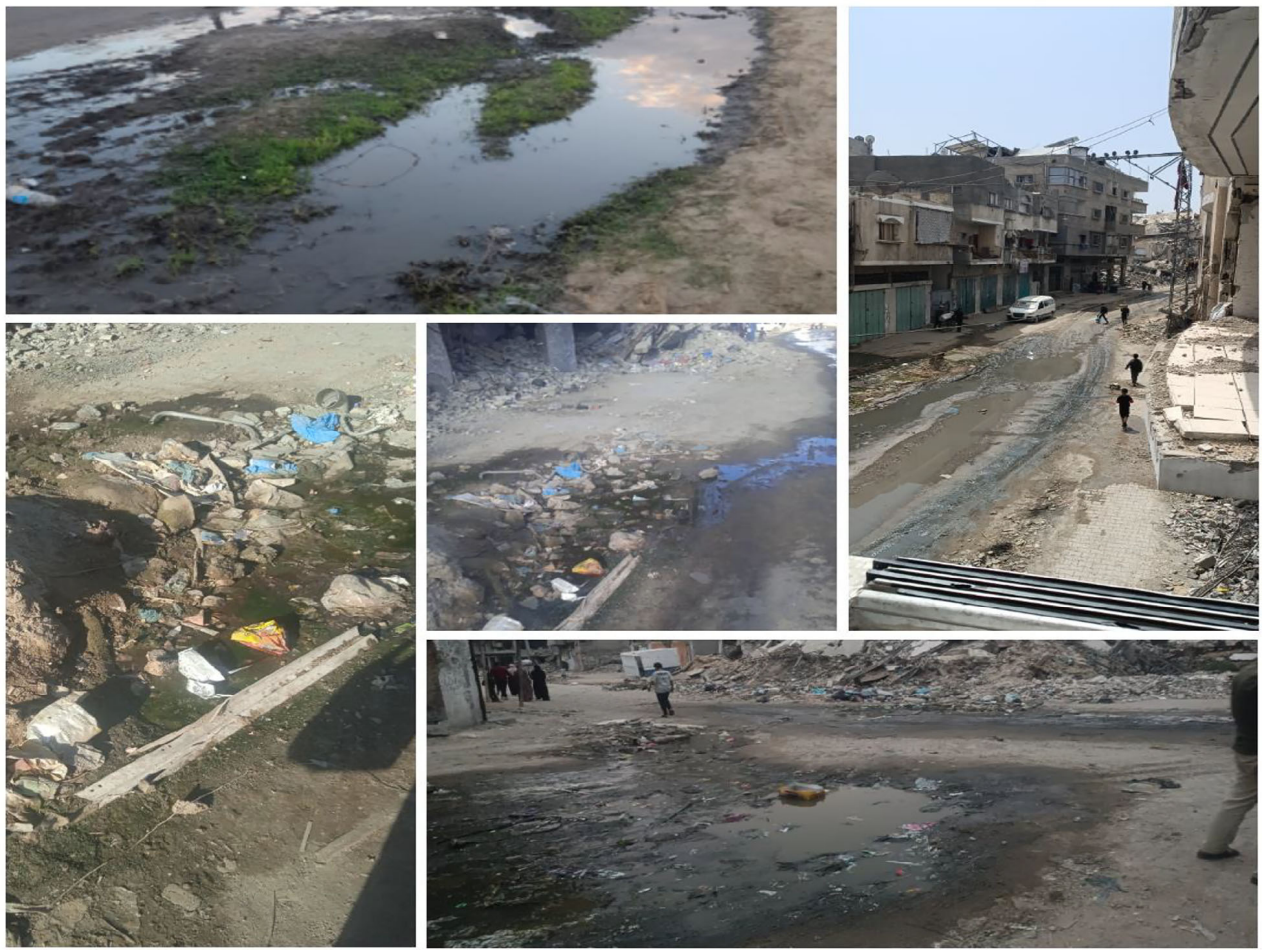
Sewage water flowing into streets and public areas within residential neighborhoods and displacement camps in Gaza during the current conflict. *Source:* Photographs by Dr. Zuhair Dardona.

If we consider the potential causes of this mosquito proliferation in Gaza during the ongoing conflict, we need not look far from the harsh reality on the ground. The systematic destruction of water and sewage infrastructure, along with the collapse of essential life‐support systems, has resulted in streets flooded with sewage and even drinking water pooling into stagnant ponds. These conditions create ideal breeding grounds for mosquitoes, fostering a fertile environment for their proliferation. Meanwhile [3], due to the destruction of infrastructure and sewage networks, sewage water flows into public streets, forming stagnant pools rich in organic matter that attract insects, particularly mosquitoes. This situation is further exacerbated by the lack of control measures and resources, alongside the near‐total destruction of residential buildings. Figure 6 illustrates the sewage water flowing between areas inhabited by displaced persons and residents in Gaza during the ongoing conflict.

Mosquito infestations present considerable public health risks, serving as vectors for numerous serious diseases, such as malaria, dengue fever, Zika virus, chikungunya, and yellow fever, all of which contribute significantly to global morbidity and mortality annually [33, 34]. Furthermore, the WHO designates mosquitoes as the most lethal animal due to their role in disease transmission, underscoring the critical need for effective control and prevention strategies to mitigate these health risks [35]. Despite the absence of officially documented outbreaks of mosquito‐borne diseases—such as malaria or dengue—in Gaza's refugee camps to date, the increasing accumulation of stagnant water, widespread insect proliferation, and ongoing deterioration of infrastructure present a significant public health concern. Local health authorities have issued warnings regarding the heightened risk of such diseases emerging should current conditions persist [36].

### 3.2 Mice Infestation

**FIGURE 7:**
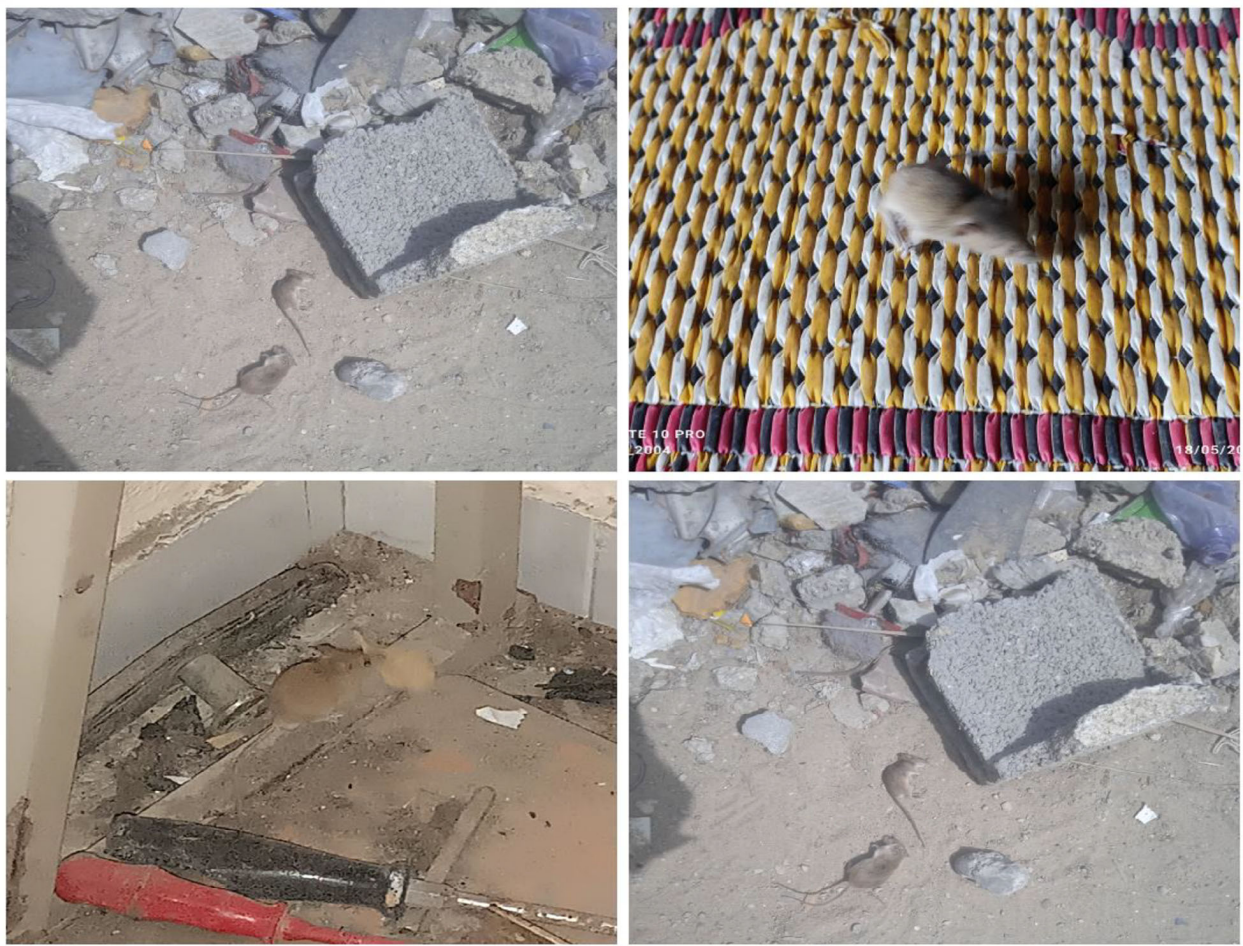
A cluster of mice in homes and streets surrounding rubble in Gaza during the ongoing conflict. *Source:* Photographs captured by Dr. Zuhair Dardona.

Extensive field investigations and photographic documentation of displacement camps, roadways, and the remnants of demolished dwellings have revealed a severe infestation of mice populations, which is a direct consequence of the ongoing conflict. These rodents have proliferated alarmingly across streets, shelters, residential areas, ruins, displacement camps, and even within the limited food storage facilities scattered throughout these environments. The mice are notably small in stature, possess elongated tails, and exhibit exceptional adaptability in concealing themselves amid rubble and debris from destroyed structures. This pervasive infestation has been consistently reported as a critical issue by both the resident population and displaced individuals within the camps. Figure 7 substantiates the widespread presence of these mice in all locations inhabited by affected populations, underscoring the significant environmental and public health challenges posed by this situation.

The critical question remains: What are the primary factors that have driven this rapid and alarming proliferation of mice in Gaza during the ongoing conflict? To answer this scientifically, it is essential first to identify the ideal environmental conditions that facilitate mice infestations. We must then apply this understanding to the current realities in Gaza amid the war and assess the extent to which these conditions coincide. Mice infestations are predominantly driven by the availability of accessible food sources, inadequate shelter, and structural vulnerabilities within human habitats [37, 38]. Improper food storage and exposed waste provide abundant nourishment, whereas cluttered and poorly maintained environments offer ideal nesting sites [38]. Structural deficiencies such as small openings and gaps facilitate easy entry, even in otherwise secure buildings [38]. Additionally, environmental factors like overhanging vegetation and disorganized outdoor materials create pathways and cover that support infestation. Human behaviors, including leaving doors or windows open and storing agricultural products near living areas, further exacerbate the problem. Coupled with the species’ rapid reproductive capacity under favorable conditions, these factors collectively contribute to the swift and extensive proliferation of mice, as consistently documented in the scientific literature [37, 38]. Furthermore, numerous published studies have firmly established a direct correlation between the accumulation of rubble and waste and increased mice infestation. These accumulations offer plentiful food resources and protective shelter, fostering optimal conditions for rodent survival and rapid reproduction [37]. Research consistently demonstrates that poor waste management practices, including exposed garbage, bulk refuse, and construction debris, significantly contribute to elevated rodent populations in both urban and residential settings [37, 39]. The enormous accumulation of rubble and rubbish in Gaza during the continuing conflict has produced an environment favorable for a significant increase in infestations by mice and other rodents. Extensive scientific literature continuously shows that the presence of significant amounts of garbage and insufficient waste management provide mice with an abundance of housing and food supplies, directly leading to population growth. In Gaza, prolonged shelling has produced over 40 million tons of debris and severely harmed waste management infrastructure, resulting in significant accumulations of uncollected rubbish in streets and refugee camps [40, 41]. Recent field investigations and official reports unequivocally indicate a substantial increase in mice and rat populations across Gaza. Moreover, both local health authorities and international organizations have extensively documented pervasive rodent activity, especially in densely populated zones characterized by the accumulation of waste and debris. Consequently, these infestations exacerbate already challenging living conditions and present serious public health threats, given rodents’ role as vectors for numerous diseases and their propensity to thrive in environments marked by inadequate sanitation and high population density [42, 43, 44]. Drawing upon the aforementioned evidence—including systematic field visits, photographic documentation, direct observations, and a comprehensive review of reports from reputable news agencies and pertinent local and international sources—the widespread proliferation of mice in the Gaza Strip amid the ongoing conflict represents a concrete and significant threat to both public health and the broader environmental context. Mice infestations present considerable environmental and public health hazards, as thoroughly documented in scientific literature and authoritative texts [45]. Environmentally, mice inflict significant structural damage by gnawing on insulation, electrical wiring, and building materials, thereby compromising the integrity of residential and public infrastructures. This damage elevates the risk of electrical fires and energy inefficiency resulting from impaired insulation. Furthermore, their burrowing and nesting behaviors accelerate the degradation of buildings and, in certain vulnerable locations, may contribute to flooding or landslides [45]. From a public health standpoint, mice are recognized as significant reservoirs and vectors for numerous zoonotic pathogens, including but not limited to leptospirosis, salmonellosis, hantavirus, lymphocytic choriomeningitis virus (LCMV), and plague, thereby representing a critical source of disease transmission to humans [46, 47, 48, 49]. In addition to the aforementioned factors, mice infestations critically impair indoor air quality through the dissemination of allergens, notably *Mus musculus* (Mus m 1), within household dust. Exposure to these allergens has been shown to precipitate and exacerbate respiratory pathologies and allergic reactions, posing heightened risks for susceptible populations, including children and individuals with asthma [47, 49]. Moreover, infestations exert significant psychological and social effects, including disrupted sleep patterns, heightened anxiety, and elevated stress levels, phenomena that are especially pronounced within densely populated urban communities [37]. Severe rodent infestations have been widely reported in Gaza's displacement camps and damaged residential areas, driven by overcrowding, infrastructural collapse, and unsanitary conditions. Documented cases of rat bites, including among children, have caused physical harm and psychological distress, with some victims requiring medical care despite limited access to treatment. Rodents pose a serious public health risk through food contamination, property damage, and potential disease transmission. Although such aggressive behavior is rare under normal conditions, the extreme environmental and humanitarian pressures appear to have intensified these incidents. Despite ongoing pest control efforts by international organizations such as UNRWA, the situation remains critical due to logistical constraints and resource shortages [50]. Overall, the ongoing conflict in Gaza has created a critical trajectory that severely threatens public health and the environment. The comprehensive collapse of essential life‐support systems—including the complete destruction of sewage networks, potable water infrastructure, garbage collection vehicles, and waste disposal sites—has compounded the crisis. Furthermore, the near‐total devastation of private and public buildings, hospitals, universities, and various institutions, coupled with the stringent blockade restricting the entry of medicines and hygiene supplies, has exacerbated the already fragile health situation. This deterioration has facilitated the spread of numerous diseases among the population. Additionally, the widespread destruction and military operations have displaced most residents of Gaza, forcing them into overcrowded camps that lack even the most basic living conditions [3, 7]. Moreover, this study encountered numerous challenges, primarily stemming from the difficult security situation prevailing during its conduct, which hindered rapid progress and comprehensive documentation of events. Additionally, limited resources and logistical constraints posed significant obstacles. Nonetheless, the researchers remained committed to ensuring clarity and accuracy in their findings to the fullest extent permitted by the available conditions and capabilities [51].

## 4 Conclusion

The current study, through a series of systematic steps including field visits, photographic documentation, reviews of authoritative websites, and extensive literature analysis, concludes that Gaza is experiencing catastrophic situation for rodent‐ and vector‐borne diseases. The destruction of all essential life‐support systems—most notably the infrastructure for sewage and potable water—has resulted in widespread flooding in streets and within displaced persons’ shelters. Additionally, the accumulation of hundreds of tons of garbage and millions of tons of rubble from demolished buildings has further exacerbated the crisis. These factors, among others, have precipitated a rapid and alarming proliferation of various insects and rodents, chiefly flies, mosquitoes, fleas, and notably mice. The absence of effective control measures, healthcare infrastructure, and adequate sanitation supplies has compounded the public health and environmental risks faced by the displaced population. This situation underscores an urgent need for comprehensive interventions aimed at restoring infrastructure, implementing robust pest control programs, and enhancing health and hygiene support to mitigate these escalating hazards.

## 5 Recommendations

This study strongly recommends, above all, the immediate cessation of hostilities to halt the ongoing humanitarian catastrophe and its far‐reaching consequences. Additionally, it advocates for the urgent provision and deployment of effective insect and rodent control materials, alongside the rehabilitation and maintenance of water supply and sewage networks to mitigate the escalating risks associated with widespread infestations. Furthermore, the study emphatically calls for the unrestricted entry of cleaning and sanitation supplies, as well as essential medicines, healthcare products, and nutritional provisions, to support the affected populations and improve overall public health conditions.

## Author Contributions

**Zuhair Dardona**: conceptualization, methodology, investigation, writing – original draft, formal analysis. **Samia Boussaa**: conceptualization, funding acquisition, writing – review and editing, validation, supervision.

## Ethics Statement

This study is based solely on observational field data and literature review. It did not involve human participants or animals; therefore, ethical approval was not required.

## Conflicts of Interest

The authors declare no conflicts of interest.

## Data Availability

All data, reviews, and Supporting Information used in this study are available from the author upon reasonable request.
